# Psychology, not technology, is our biggest challenge to open digital morphology data

**DOI:** 10.1038/s41597-019-0047-0

**Published:** 2019-04-26

**Authors:** Christy A. Hipsley, Emma Sherratt

**Affiliations:** 1 School of BioSciences, University of Melbourne, Parkville, VIC 3010 Australia; 2 Museums Victoria, GPO Box 666, Melbourne, VIC 3001 Australia; 3 School of Biological Sciences, The University of Adelaide, Adelaide, SA 5005 Australia

**Keywords:** Publishing, Taxonomy, Research data

## Abstract

The past two decades have seen a revolution in digital imaging techniques for capturing gross morphology, offering an unprecedented volume of data for biological research. Despite the rapid increase in scientific publications incorporating those images, the underlying datasets remain largely inaccessible. As the technical barriers to data sharing continue to fall, we face a more intimate, and perhaps more complicated, obstacle to open data – the one in our minds.

Open data – data that can be freely used, reused and redistributed by anyone – is driven by the digitization of information into an easily sharable form. While the technical aspects of this process have improved dramatically over the past decade^1–3^, many scientific fields have yet to establish an open data standard^4^. This lack of consensus has been particularly troubling for the systematics community, which increasingly relies on digital imaging techniques like X-ray computed tomography (CT) and magnetic resonance imaging (MRI) to visualize and compare biological forms (Fig. 1a). The resulting datasets, composed of hundreds or thousands of individual images, can be digitally reconstructed as high-fidelity 3D models that act as virtual avatars of the physical specimens, thereby protecting often rare and vulnerable material. These models can be copied and analysed multiple times, allowing simultaneous use by researchers all over the world. They also provide computational substrate for downstream analyses, making access to the digital files critical for the transparency, reproducibility, and continuity of scientific discovery.

**Fig. 1:**
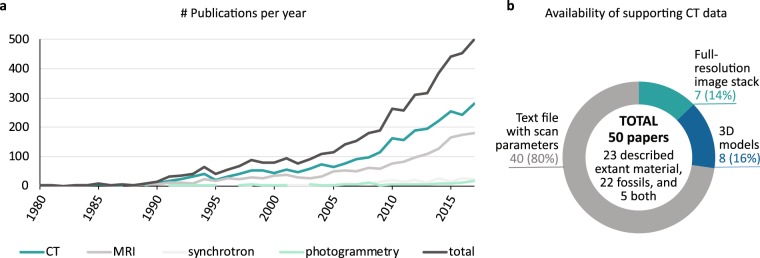
Increasing use and poor availability of digital morphology data in systematics research. (**a**) Number of publications per year retrieved through Web of Science’s Science Citation Index Expanded using the topic search terms: x-ray comput* tomograph* OR CT; magnetic resonance imag* OR MRI; synchrotron; and photogrammetr*. Articles were limited to Web of Science categories Anatomy Morphology, Evolutionary Biology, Paleontology, and Zoology. Results for 2018 are not shown. Only three articles prior to 1980 were recovered. (**b**) Availability of underlying CT datasets supporting 50 papers published in the past 5 years in *Anatomical Record*, *Journal of Anatomy*, *Journal of Morphology*, *Journal of Vertebrate Paleontology*, and *Zoological Journal of the Linnean Society* (10 papers each). These journals were among the top 10% of publishers including CT data recovered in our literature search.

Although most scientists agree on the benefits of open data^3,5^, issues surrounding data sharing management^6^, curation^7^, enforcement^8^, and associated costs^9^ persist. These concerns are largely technical, meaning their solutions lie in improved technology and support to guide producers through the data sharing experience. Practical aids for many steps are already in place, including data management guidelines^10^, repository registries (e.g., www.re3data.org), searchable policy databases, and free (e.g., figshare, Zenodo) or institutional (e.g., Harvard Dataverse) data sharing platforms. Some journals like *Evolution* and *Ecology Letters* also offer data archiving services, making digital images accessible for peer review and providing automatic deposition upon acceptance. Moreover, a large group of leading biologists and paleontologists^11^ recently proposed a set of minimum standards and best practices for sharing 3D digital morphology data via online repositories, making it easier than ever to achieve the scientific ideal.

While such open-access policies would have tangible benefits for the scientific community and society at large in terms of maximized investment in research funding and infrastructure, enhancement of biodiversity collections, public outreach, and education, they are generally not followed^1,5,8^. Many scientists fear a loss of control over their hard-earned data once they go public, the effects of which are exacerbated by unequal access to resources (e.g., research provisions, financial support, infrastructure)^12^ and lack of personal incentives^13^. This reluctance is evident in our survey of the systematics literature, in which only 7 of 50 papers (14%) incorporating CT images in the past five years made their data publicly available^14^ (Fig. 1b). This failing in more recent publications is not due to lack of appropriate venues, since online repositories for ‘Big Data’ have been available since the 2000s (e.g., Dryad, Morphobank), often free of charge with options for licensing and citability.

What then stands in our way of achieving an open data standard? We surveyed over 100 researchers in the systematics community about the discrepancy between recommended data sharing standards and current practices, and found that psychology, not technology, remains our biggest obstacle.

## Perceived loss of power through sharing

Digital imaging is a labor-intensive process that incorporates subjectivity at every step, from choice of acquisition method, instrument, and sample preparation, to software implementation, visualization, and post-processing^15^. Although most respondents were willing to publish their digital morphology data either directly (42%) or following embargo (56%), many expressed concerns over proper attribution and how their data would be used^14^. Lack of reward or recognition as well as fear of being scooped, poached, or misrepresented^1,13^ are strong deterrents to sharing, the impacts of which are larger for early-career researchers invested in single datasets^5,16^. Risk of ‘losing face’, or that others will find errors in one’s work, is another mental barrier to publishing complex information that is often viewed as private intellectual property^17^.

For others, willingness to share digital morphology data depended on the amount and rarity of imaged specimens (few vs many, fossils vs common species), type (3D models, image stacks) and receiving parties (individuals vs corporations)^14^. Communication with the author and publisher latency terms are suggested to mitigate these factors, by providing scientists more proprietary control and adequate time to exploit their own data. However, what those terms might be regarding a reasonable time frame (months vs years) and request (number of specimens) is still unclear. Co-authorship demands for published morphology data are still common (8–35% of requests^14^), even when the proprietary controller did not pay for data acquisition or when a long time has passed since the images were acquired. In one case, a respondent’s refusal to add a ‘gratuitous author’ to their paper for use of published CT images resulted in them scanning the same materials again – a perceived waste of time, money and effort.

In spite of these uncertainties, the majority of respondents have asked (62%) or have been asked (62%) to share digital morphology data^14^. As these images are typically generated through multi-institutional efforts and contribute to diverse analyses (e.g., species descriptions, geometric morphometrics, biomechanical modelling), promotion of an open data culture in favor of increased transparency, collaborative opportunities, and public recognition should help to dissuade concerns over individual losses.

## Would but can’t, could but don’t

**Fig. 2:**
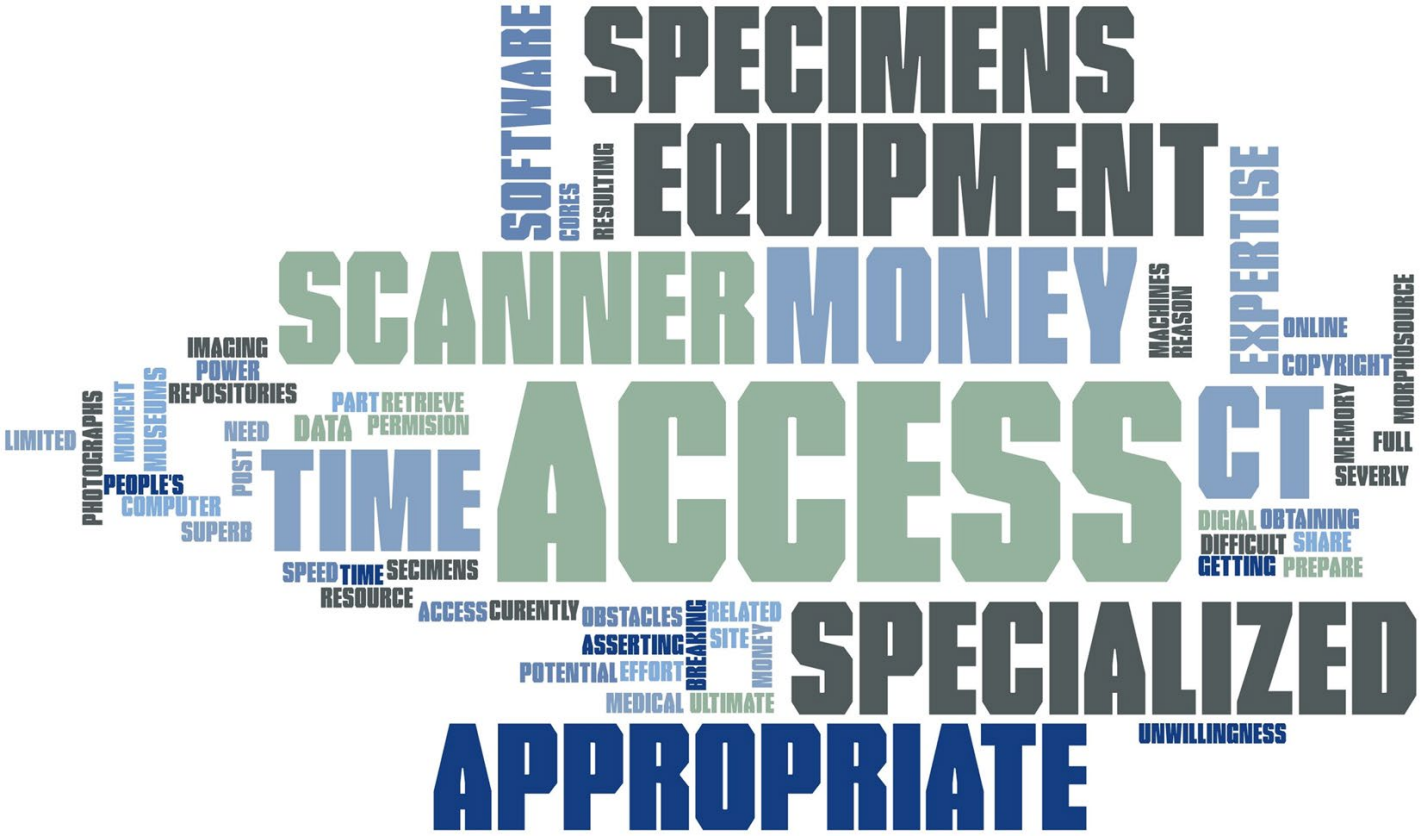
Word cloud of 117 survey responses to the question ‘What do you see as the biggest obstacles to obtaining digital morphology data?’.

In response to the question ‘What do you see as the biggest obstacles to obtaining digital morphology data?’, access to appropriate specimens, equipment, time, and money emerged as the most common replies (Fig. 2). Social and economic barriers are known to discourage sharing, with the level of effort a researcher is willing to exert to publish their dataset varying according to available resources^17^. Imaging techniques like CT and MRI can be costly, which in addition to specialized equipment require trained personnel, customized hard- and software, and dedicated facilities with monitored safety protocols. Furthermore, specimen loans for imaging often require museum visits or other personal interactions with managing staff – an endeavor that is likely facilitated when the borrower is from a prominent lab with a known head.

Asymmetries in financial support, reputation, and other academic resources not only affect the ability to publish digital morphology data for individuals vs ‘big labs’, but also for researchers in developed vs lesser developed regions. Technological capabilities to integrate data into open platforms differ markedly in low/middle-income countries (LMICs), where power-cuts, slow internet connections, and out-of-date computing facilities can limit data sharing activities for both generators and users^14^. Targeted incentives like fee waivers and extended embargo times for dataset deposition are already emerging^12^, although further support initiatives need also focus on minimizing data sharing disincentives imposed by low-resource environments (e.g., connectivity). Similar to early career researchers, fear of being scooped is another major concern for scientists in LMICs, because of the lower funding and longer time it takes to publish there^12^.

Although our respondents are likely from high-income countries, the majority (60%) were non-tenured (students, postdocs), and of those over half paid for generation of their digital morphology data^14^. While some institutes have equipment and policies for in-house imaging, others offer reduced collaborator rates in exchange for co-authorship. This practice has left a bad taste in the mouth of some scientists, who view such attribution as gratuitous and monopolistic of the digital data. At the same time, established researchers with exclusive access to specimens and equipment are not necessarily more likely to publish their datasets than those with fewer resources. The recently proposed best practices^11^ for sharing 3D digital morphology data in support of published papers should improve this disparity, although data unassociated with peer-reviewed articles may languish indefinitely on hard drives and personal cloud stores due to issues of trust (scooping), resources, and (dis)incentives^12^.

## Share more to get more

Given observed variation in researchers’ willingness to share digital morphology data, creating a system that aligns social and individual incentives is an obvious step towards leveling the playing field^1^. Increased visibility, public recognition, and downstream collaborations are all recognized benefits of open data, especially for junior scientists and those in LMICs^12,13^. Translating those benefits into quantifiable impacts provides an alternative to conventional metrics (i.e., citations, *h*-index)^5^, which can take years to accumulate and ignore new types of communication. So-called ‘altmetrics’ aim to do just that by measuring how scientific content is shared, used, and discussed across social media and publisher sites. Incorporation of altmetrics for published datasets into researcher CVs, grant applications, and progress reports would allow individuals to capitalize on their data sharing behaviours, while providing new opportunities for networking and scientific progress. Indeed, many of our respondents indicated that acknowledgement was an important incentive to sharing digital morphology data, preferably as citable objects^14^.

Eventually, some believe, there will be so much data available that ownership will no longer be an issue^13^. In the past decade alone, the number of publications incorporating digital morphology images into systematics research have more than tripled, with over half of those using CT (Fig. 1a). Considering a single dataset including image stacks, 3D models, and relevant metadata can reach over 100 GB per specimen^11^, management of CT and other digital files poses a serious challenge. Many argue that standardization of file types and a single, centralized repository would alleviate proprietary concerns, by streamlining dataset preparation, providing DOIs or other citable reference codes, and maintaining permanent storage of the specimen images^11,14^. The public repository GenBank presents a similar model, in which genetic sequences are annotated, quality-controlled, and permanently accessioned, whether or not they underlie published studies. This repository now constitutes one of the single most influential databases in biological sciences, with an exponential growth rate and over 285 billion nucleotide bases^18^. Reaching an analogous threshold in digital morphology where everyone experiences positive returns on their open data investments will require individual buy-in and clear institutional and journal-mandated policies.

## Whose responsibility is it anyway?

*‘Publicly funded research should result in shared data’*^14^. This is a common viewpoint among researchers^13^, and one that is advocated by most major scientific funding agencies. The National Science Foundation (NSF) states that ‘*Investigators are expected to share with other researchers, at no more than incremental cost and within a reasonable time, the primary data, samples, physical collections and other supporting materials created or gathered in the course of work under NSF grants*’ (https://www.nsf.gov/bfa/dias/policy/dmp.jsp). Since 2011, NSF proposals are required to include a data management plan with procedures for deposition in an appropriate repository, regardless if those data support a published paper.

In contrast to these expectations, we found that of the 16 NSF-funded papers we reviewed, only four made their complete CT datasets available as full-resolution image stacks (e.g., TIFFs), while another three provided project links to online repositories but failed to deposit their data there^14^. These observations are irrespective of publisher, since all five of the surveyed journals encourage authors to archive their data while providing support on how to do so. For example, Wiley (producer of *Anatomical Record*, *Journal of Anatomy* and *Journal of Morphology*) hosts an online Author Compliance Tool (https://authorservices.wiley.com/author-resources/Journal-Authors/open-access/author-compliance-tool.html), providing users with information on data sharing policies relevant to their specific funder, institution, and journal, as well as details on mandated repositories, when available. From 2018, Taylor & Francis, home to *Journal of Vertebrate Paleontology*, also introduced new policies on data sharing with links to searchable registries of discipline-specific and generalist repositories, in addition to SHERPA Juliet (http://v2.sherpa.ac.uk/juliet/), an online database of funders’ open-access mandates.

Despite increasingly clear guidelines on dataset deposition, many authors feel they do not have the time and most contact the individual and not the repository to request digital morphology data^14^. Among our respondents, at least 60% of such requests were granted, compared to much lower success rates from surveys in other fields (e.g., 10% in medicine^19^, 15% in psychology^20^). Journals themselves should have a vested interest in enforcing open data mandates, as papers with publicly available datasets receive more citations than those without – a benefit that persists for many years after the original author has finished exploiting their data^21^. Peer reviewers of submitted manuscripts also play an important role in policing data compliance, by examining supporting material and ensuring that credit is given to the original author(s) in cases of reuse^11^. The increasing availability of free and open source software packages for visualizing 3D volumes (e.g., ImageJ, Drishti, CTVox, 3D Slicer) should facilitate this process, particularly if they can be integrated into the journal’s editorial platform.

Institutional policy on digital data ownership (e.g., museums vs researchers) and dissemination is another area in need of consensus, since the majority of our respondents claimed that their workplace either lacked such policy entirely (68%), or that they were unsure if it existed (13%)^14^. Many museums limit third-party sharing of image data, which are viewed as digital resources analogous to the physical specimens they represent. For example, the American Museum of Natural History requires researchers to sign a user agreement for CT data based on their collections, allowing data archiving in public repositories while retaining copyright ownership of ‘images, scans, raw data, pre-processed data, rendered data, and all derivatives of its specimens, including photos, molds, x-rays, and printed casts’ (www.amnh.org/our-research/paleontology/visitors/3d-scanning). This distinction between data archiving as a primary requirement and data sharing is important, since confusion between the two can result in poor data management plans at best, and permanent loss of specimen images at worst. In either case, organizational support including legal regulations (i.e., copyrights) and data management is a significant predictor of knowledge sharing behaviour^17^, making this often-ambiguous element ripe for reform.

Regardless of remaining challenges, we are optimistic about the future of open data. A *Nature* News Feature published during the revision phase of this Comment (‘The fight for control over virtual fossils’)^22^ found that among 59 paleontology papers published from 2017–2018 involving 3D imaging, 31% made their data publicly available – more than double that found in our survey of papers from the past 5 years including fossil and modern specimens. We interpret this difference in two ways, 1) that paleontologists and museum curators are more likely to make their fossil specimen data available, probably to avoid further handling of sensitive material, and 2) that data openness is improving over time. They also surveyed a wider range of journals (47 compared to our 5), some of which may have stricter policies regarding data availability. Nevertheless, this trend indicates that the best practice guidelines outlined by Davies *et al*.^11^ are making an impact, at least in paleontology. Other professional societies are also beginning to formalize their own data sharing agreements, like the American Association of Physical Anthropologists for images of humans and non-human primates (www.nsf.gov/awardsearch/, Award #1826885), suggesting expanded access to digital files across multiple disciplines in the future.

Ultimately, data sharing involves a commitment from individuals in the scientific community to fully realize the potential of open digital morphology. Some players are embracing this transition, for example the NSF-funded initiative oVert (www.floridamuseum.ufl.edu/science/overt/) to provide free digital 3D anatomy for 20,000 fluid-preserved animals. Scanned images are made available through the online repository MorphoSource (www.morphosource.org), with corresponding data linked to iDigBio (www.idigbio.org), an aggregator of specimen information from natural history collections. Under this system, curators and other contributors have the option to oversee ‘virtual loans’ of their specimen data and receive regular updates on image use. This effort to create an integrated computing environment with flexible controls could free up time and money of public museums, whose staff are already tasked with caring for the physical specimens. It would also encourage development of automated pipelines for anatomical phenotyping, a field that holds much promise for digital datasets too large to sift through by trained experts^23^.

Finally, the FAIR principles encouraged by *Scientific Data* and other journals state that all research objects should be Findable, Accessible, Interoperable and Reusable (FAIR)^24^. As our community navigates the shift to FAIR data standards, we as stakeholders stand to benefit the most from overcoming mental barriers to open data and prioritizing long-term stewardship of our digital resources. Because ‘data available upon reasonable request’ is no longer a reasonable option.
